# Group II innate lymphoid cells and microvascular dysfunction from pulmonary titanium dioxide nanoparticle exposure

**DOI:** 10.1186/s12989-018-0280-2

**Published:** 2018-11-09

**Authors:** Alaeddin Bashir Abukabda, Carroll Rolland McBride, Thomas Paul Batchelor, William Travis Goldsmith, Elizabeth Compton Bowdridge, Krista Lee Garner, Sherri Friend, Timothy Robert Nurkiewicz

**Affiliations:** 1 Department of Physiology and Pharmacology, West Virginia University School of Medicine, 64 Medical Center Drive, Robert C. Byrd Health Sciences Center - West Virginia University, Morgantown, WV 26505-9229 USA; 2 Toxicology Working Group, West Virginia University School of Medicine, Morgantown, WV USA; 3 National Institute for Occupational Safety and Health, Morgantown, WV USA

**Keywords:** Engineered nanomaterials, Titanium dioxide nanoparticles, Microcirculation, Innate lymphoid cells, Inflammation

## Abstract

**Background:**

The cardiovascular effects of pulmonary exposure to engineered nanomaterials (ENM) are poorly understood, and the reproductive consequences are even less understood. Inflammation remains the most frequently explored mechanism of ENM toxicity. However, the key mediators and steps between lung exposure and uterine health remain to be fully defined. The purpose of this study was to determine the uterine inflammatory and vascular effects of pulmonary exposure to titanium dioxide nanoparticles (nano-TiO_2_). We hypothesized that pulmonary nano-TiO_2_ exposure initiates a Th2 inflammatory response mediated by Group II innate lymphoid cells (ILC2), which may be associated with an impairment in uterine microvascular reactivity.

**Methods:**

Female, virgin, Sprague-Dawley rats (8–12 weeks) were exposed to 100 μg of nano-TiO_2_ via intratracheal instillation 24 h prior to microvascular assessments. Serial blood samples were obtained at 0, 1, 2 and 4 h post-exposure for multiplex cytokine analysis. ILC2 numbers in the lungs were determined. ILC2s were isolated and phosphorylated nuclear factor kappa-light-chain-enhancer of activated B cells (NF-ĸB) levels were measured. Pressure myography was used to assess vascular reactivity of isolated radial arterioles.

**Results:**

Pulmonary nano-TiO_2_ exposure was associated with an increase in IL-1ß, 4, 5 and 13 and TNF- α 4 h post-exposure, indicative of an innate Th2 inflammatory response. ILC2 numbers were significantly increased in lungs from exposed animals (1.66 ± 0.19%) compared to controls (0.19 ± 0.22%). Phosphorylation of the transactivation domain (Ser-468) of NF-κB in isolated ILC2 and IL-33 in lung epithelial cells were significantly increased (126.8 ± 4.3% and 137 ± 11% of controls respectively) by nano-TiO_2_ exposure. Lastly, radial endothelium-dependent arteriolar reactivity was significantly impaired (27 ± 12%), while endothelium-independent dilation (7 ± 14%) and α-adrenergic sensitivity (8 ± 2%) were not altered compared to control levels. Treatment with an anti- IL-33 antibody (1 mg/kg) 30 min prior to nano-TiO_2_ exposure resulted in a significant improvement in endothelium-dependent dilation and a decreased level of IL-33 in both plasma and bronchoalveolar lavage fluid.

**Conclusions:**

These results provide evidence that the uterine microvascular dysfunction that follows pulmonary ENM exposure may be initiated via activation of lung-resident ILC2 and subsequent systemic Th2-dependent inflammation.

**Electronic supplementary material:**

The online version of this article (10.1186/s12989-018-0280-2) contains supplementary material, which is available to authorized users.

## Introduction

Reproductive toxicity is increasingly becoming recognized as a critical aspect of ENM safety. However, the effects of ENM exposure on overall reproductive health have only recently been addressed and become a focus of intense study by numerous groups [1, 2]. As with other observations of systemic biologic effects after pulmonary ENM exposures, reproductive effects are equally susceptible but poorly understood. Our group has previously reported that nano-TiO_2_ inhalation is associated with uterine microvascular dysfunction [3], potentially deleterious epigenomic alterations [4], and cognitive deficits in maternally exposed progeny [5]. While these studies highlight the importance of identifying the maternal effects of ENM exposure, the mechanisms linking such exposures to these negative reproductive and developmental outcomes have yet to be fully elucidated.

In the last decade, innate lymphoid cells (ILC) have emerged as a novel population of tissue-specific effector cells with the ability to initiate and regulate the innate and adaptive branches of the immune system [6, 7]. ILCs are now divided into 3 distinct groups according to the pattern of cytokine secretion; Group 1 ILC (ILC1) are predominantly tissue-resident cells capable of secreting interferon gamma (IFN- γ) in the liver, gut, spleen, skin, peritoneum, and salivary gland [8]. The role played by ILC1 in various immunological conditions remains unclear but is currently under investigation. Group 2 ILCs (ILC2, also known as nuocytes, natural helper cells) secrete IL-4, IL-5, IL-9, and IL-13 in response to damage-associated molecular patterns or “alarmins” and have been implicated in the immune response to parasitic infections and in allergic airway inflammation [9] to several environmental and anthropogenic agents [10, 11]. Lastly, group 3 ILCs (ILC3) produce IL-22 and or IL-17, are enriched at mucosal sites, contribute to the maintenance of the intestinal barriers, and may play a significant role in the promotion of the inflammatory response and etiology of inflammatory bowel diseases [12, 13]. One of the most important alarm signals secreted by cells is interleukin-33 (IL-33) [14]. IL-33 is a member of the IL-1 family of cytokines expressed by both non-immune cells such as epithelial, endothelial, smooth muscle cells, and fibroblasts [15], as well as immune effector cells including macrophages and dendritic cells. Its role in directing the inflammatory response following pulmonary exposure to carbon nanotubes [16–18], and ozone [19] have been previously reported. IL-33 has been demonstrated [20] to induce the release of Th2 cytokines by immune cells including ILC2s in asthmatic patients [21], and to induce airway hyperresponsiveness and increased pulmonary resistance [22, 23].

In view of the increasing number of studies indicating a crucial role for ILCs in the initiation of the acute inflammatory response to environmental agents, the goal of this work was to provide initial evidence of their potential involvement in the response to pulmonary ENM exposure. Therefore, the purpose of this study was threefold. First, we identified the inflammatory and uterine microvascular effects associated with acute pulmonary nano-TiO_2_ exposure. Second, we determined the possible role played by ILC in the immune response mounted to acute pulmonary nano-TiO_2_ exposure. Lastly, the microvascular and systemic effects of treatment with an IL-33 antibody (a key activator of ILC2) were determined. We hypothesized that pulmonary nano-TiO_2_ exposure initiates a Th2 inflammatory response mediated involving IL-33 and potentially involving ILC2, which may be associated with a systemic impairment in uterine microvascular reactivity.

## Results

### SEM images and mass spectrometry of Nano-TiO_2_

**Fig. 1:**
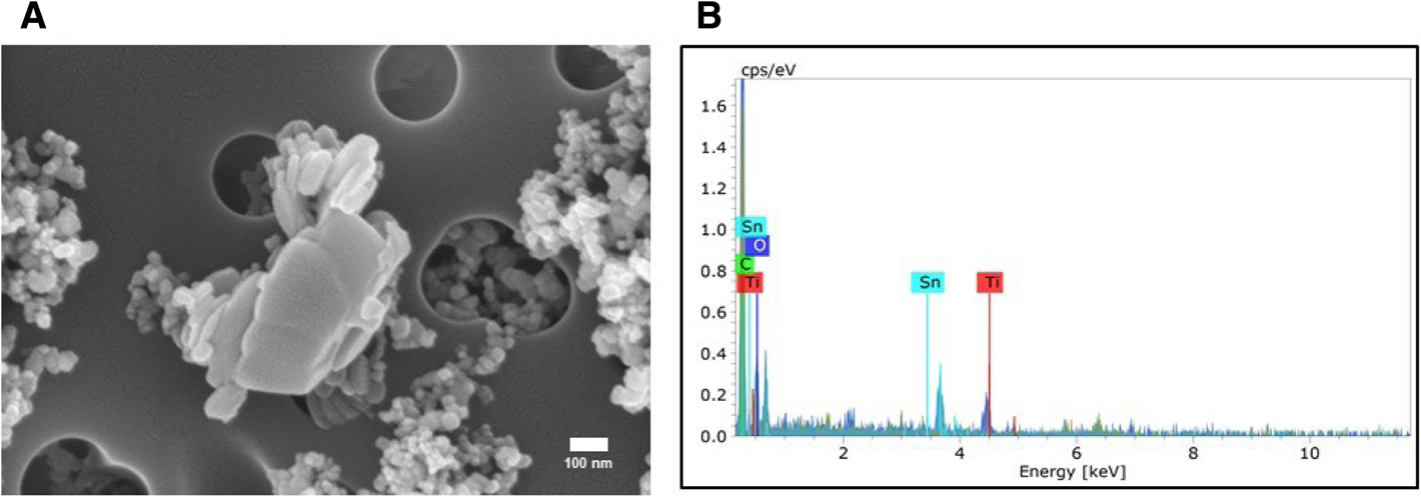
Characterization of Nano-TiO_2_. (**a**) SEM image and (**b**) Energy dispersive spectroscopy showing elemental composition of the nano-TiO_2_ suspension used in this study

Figure 1 shows field-emission scanning electron microscope images (Hitachi S4800, Tokyo, Japan) of the nano-TiO_2_ suspension used for this study. As seen in Fig. 1a, the suspended particles present significant agglomeration. Figure 1b shows the elemental composition of the nano-TiO_2_ suspension (Bruker, Billerica, MA), indicating the prevalent presence of titanium.

### Animal and vessel characteristics

No significant changes were observed in age, mean arterial pressure (MAP), heart rate and body weight between control and exposure groups **(**Table 1**)**. Additionally, radial arteriolar inner and outer diameter, tone, passive diameter, wall thickness, wall to lumen ratio and calculated wall tension were not affected by nano-TiO_2_ exposure and treatment with an anti-IL-33 antibody **(**Table 2**)**. From these results we can infer that acute nano-TiO_2_ exposure does not alter vascular tone or the balance of its contributing influences.

**Table 1:** Animal characteristics

	N	Age (weeks)	weight (grams)	Heart Rate (bpm)	Map (mm Hg)	Systolic Blood pressure (mm Hg)	Diastolic Blood Pressure (mm Hg)
Control	13	14 ± 1	232 ± 5	319 ± 13	85 ± 3	103 ± 4	73 ± 2
Exposed	14	15 ± 1	249 ± 12	330 ± 9	86 ± 4	110 ± 6	78 ± 4

Table showing characteristics of control (*N* = 13) and exposed groups (*N* = 14). Values shown are mean ± SEM. Statistics were analyzed with two-way ANOVA (*P* ≤ 0.05), * Sham control group vs. nano-TiO_2_ exposed groups

**Table 2:** Arteriolar characteristics

	n	Inner diameter (μm)	Outer diameter (μm)	Tone(%)	Passive diameter inner (μm)	Passive diameter outer (μm)	Wall Tension (Newton/meter)
Control	18	98 ± 5	152 ± 14	26 ± 7	152 ± 9	199 ± 15	0.33 ± 0.1
Exposed	16	102 ± 11	164 ± 13	21 ± 4	159 ± 15	207 ± 17	0.34 ± 0.1
Exposed + anti-L-33	12	97 ± 11	158 ± 19	21 ± 4	140 ± 14	187 ± 26	0.31 ± 0.6

Table showing characteristics of control and exposed radial arterioles (*n* = 12–18). All vascular assessments were performed 24 h post-exposure. Values shown are mean ± SEM. Statistics were analyzed with two-way ANOVA (P ≤ 0.05), * Sham control group vs. nano-TiO_2_ exposed groups

### Acute plasma and BALF cytokine secretion patterns following pulmonary Nano-TiO_2_ exposure

**Fig. 2:**
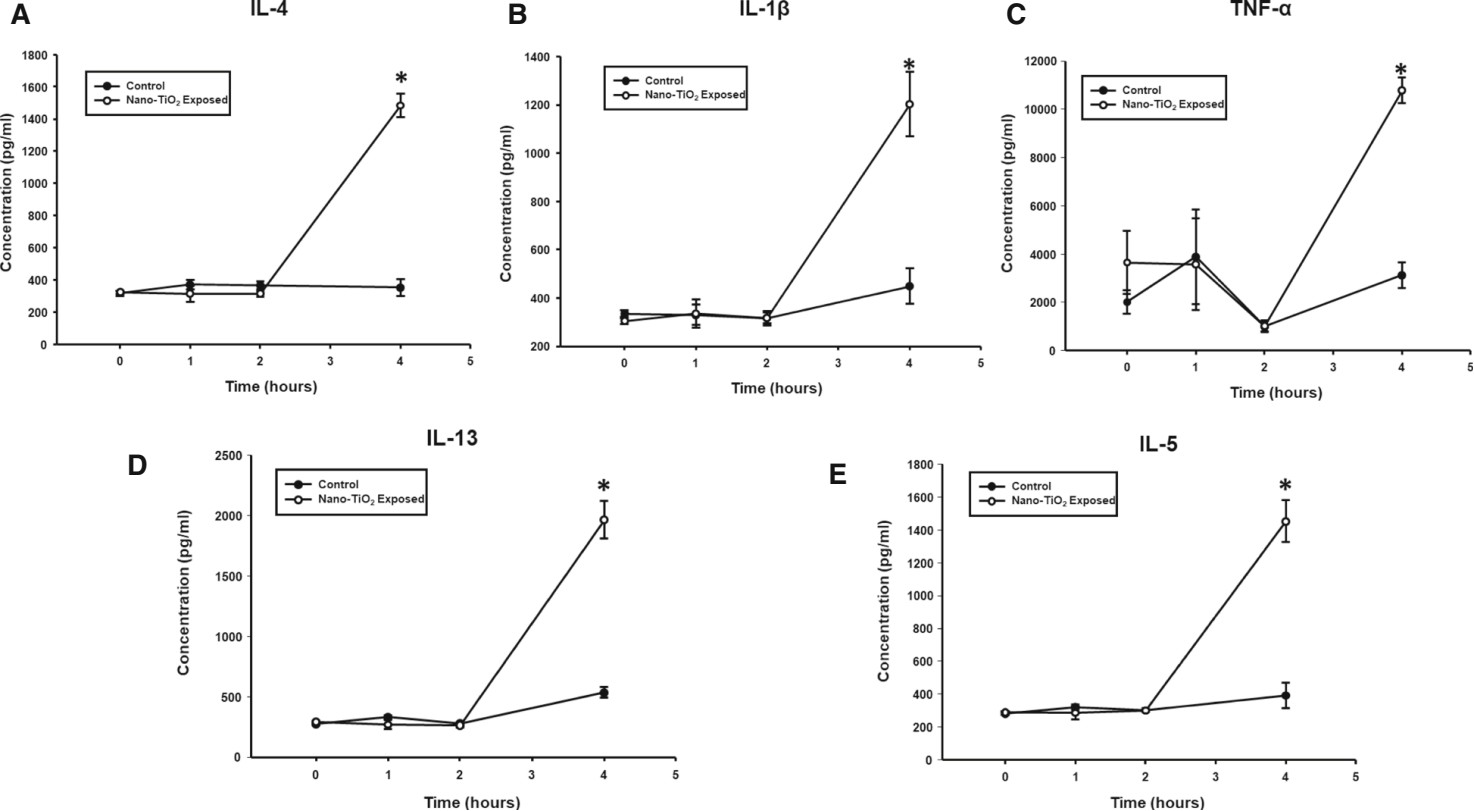
Nano-TiO_2_ exposure increases T-Helper type II cytokines 4 h post-exposure. Multiplex cytokine analysis showing concentrations of IL-4 (**a**), IL-1β (**b**), TNF-α (**c**), IL-13 (**d**) and IL-5 (**e**). Serum samples were obtained at 0, 1, 2 and 4 h post-exposure via tail-vein puncture (*N* = 6). Statistics were analyzed with two-way ANOVA (*P* ≤ 0.05), * Sham control group vs. nano-TiO_2_ exposed groups

A time-course study was initially performed to identify the temporal effect of nano-TiO_2_ on cytokine secretion. Plasma samples were obtained at 0,1,2, and 4 h following nano-TiO_2_ exposure by tail vein puncture. Figure 2 shows the results of the multiplex analysis. No differences existed at time 0, 1 or 2 h post-exposure. However, significant differences were detected 4 h post-exposure in plasma levels of the pro-inflammatory cytokines IL-4, IL-5, IL-13, TNF-α, IL-1β, while no alterations in the cytokines IFN-γ, KC/GRO, TNF-α, IL-10, IL-6, MCP-1, TIMP-1, Lipocalin-2, and TSP-1 were noted *(Data not shown)*.

**Fig. 3:**
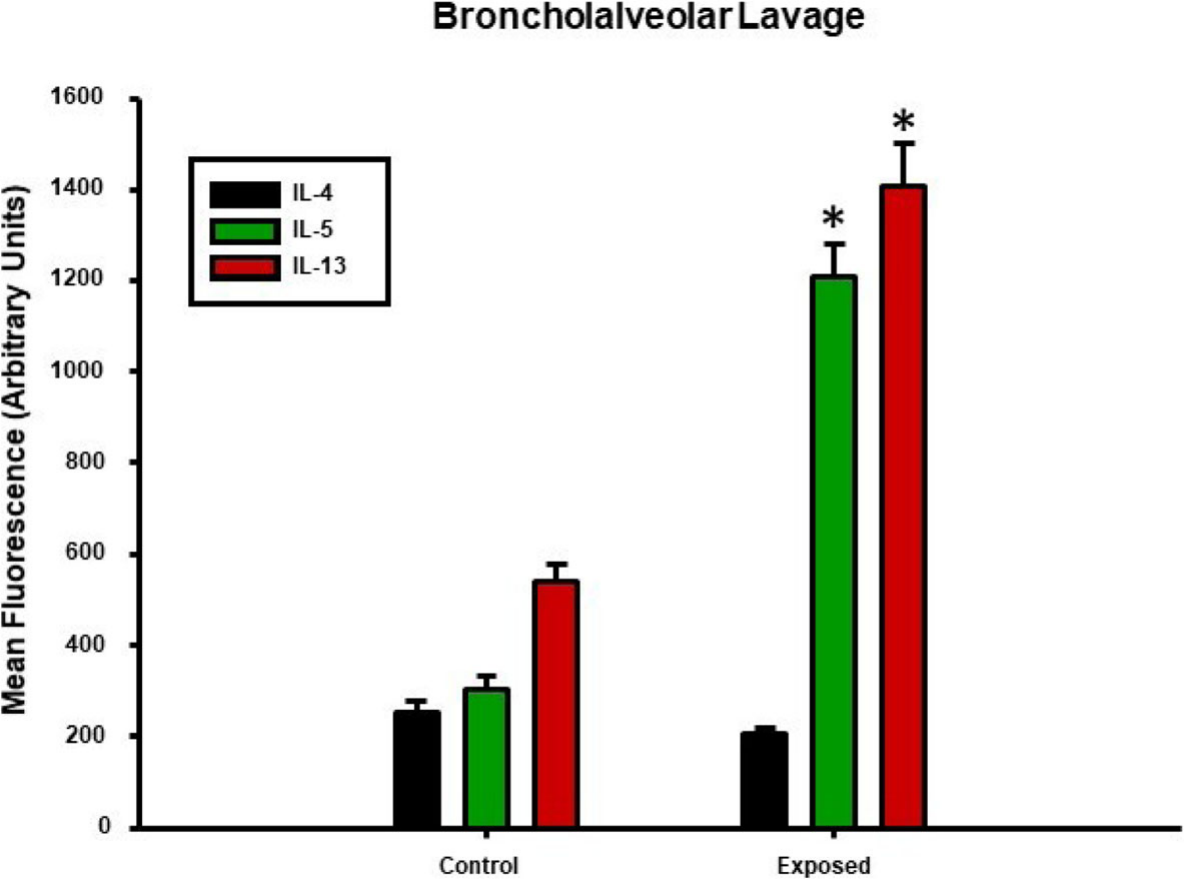
Nano-TiO_2_ exposure also increases T-Helper type II cytokines 4 h post-exposure in bronchoalveolar lavage fluid (BALF). Multiplex cytokine analysis showing concentrations of IL-4, IL-5, IL-13 in BALF from exposed and control animals 4 h post-exposure (*N* = 6). Statistics were analyzed with two-way ANOVA (*P* ≤ 0.05), * Sham control group vs. nano-TiO_2_ exposed groups

In order to confirm the results observed in the plasma, the cytokine levels in the BALF were also measured 4 h post-exposure. Multiplex analysis of the BALF **(**Fig. 3**)** also showed an almost 5-fold increase in IL-5 and a 6-fold increase in IL-4, IL-13, TNF-α, and IL-1β, while no changes were seen in the other cytokines *(Data not shown)*. Based on the collective cytokine profile associated with this exposure paradigm, the results provide evidence that acute pulmonary nano-TiO_2_ exposure may trigger a T-Helper cell type 2 (Th2) response beginning 4 h post-exposure.

### IL-33 secretion by lung epithelial cells pre- and post- exposure and following treatment with an anti-IL-33 antibody

**Fig. 4:**
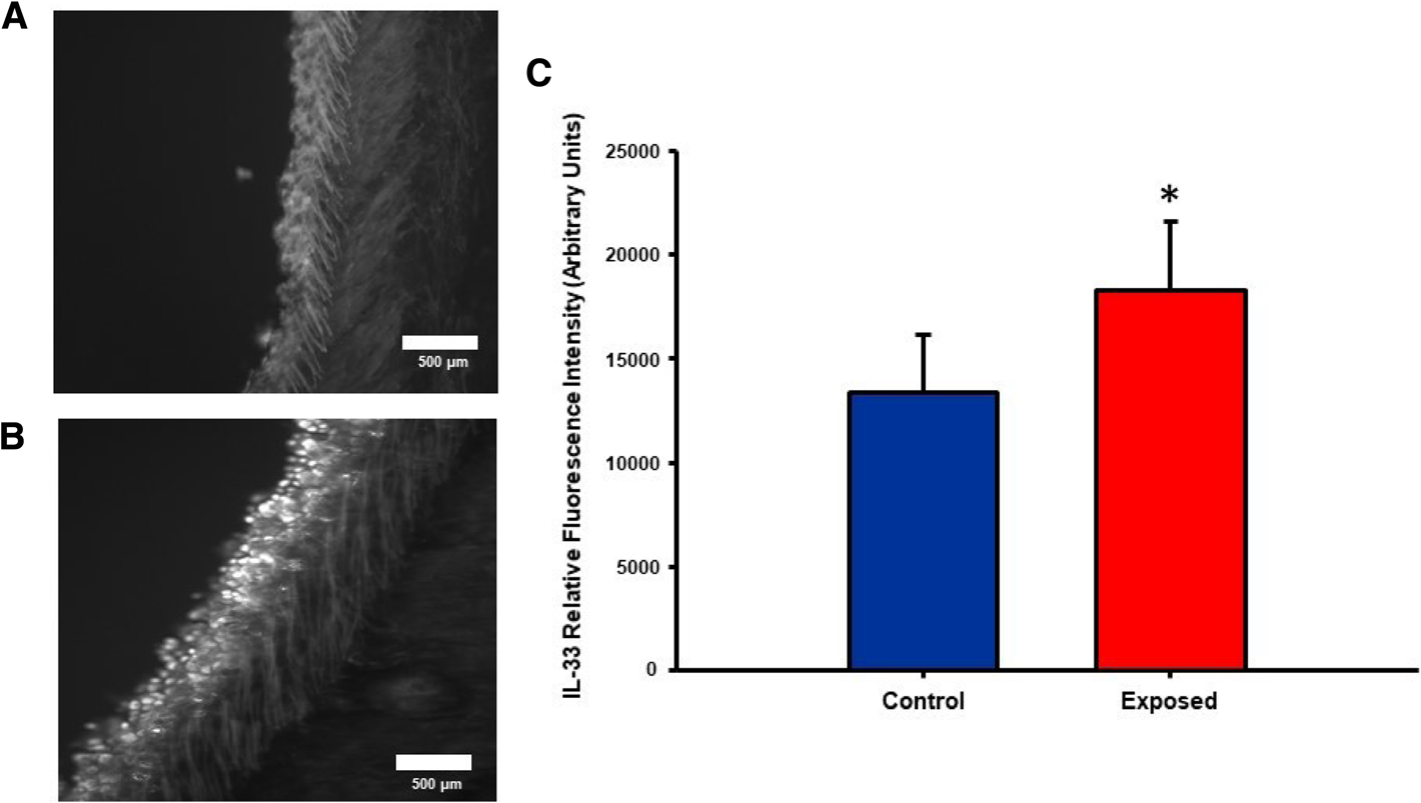
Nano-TiO_2_ exposure is associated with an increase in pulmonary interleukin-33 levels. Lung sections from control (**a**) and nano-TiO_2_ exposed (**b**) animals obtained 4 h post-exposure were stained for interleukin-33. Fluorescence was achieved by staining tracheal sections with an anti-IL-33-FITC conjugated antibody. Relative fluorescence is shown in (**c**) tagged antibody (N = 6–7). Statistics were analyzed with two-way ANOVA (*P* ≤ 0.05), * Sham control group vs. nano-TiO_2_ exposed groups

**Fig. 5:**
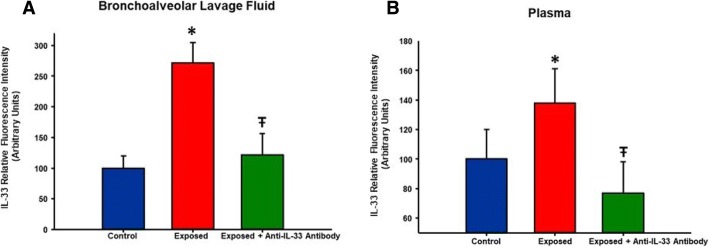
Pre-treatment of nano-TiO_2_ – exposed animals with an anti-IL-33 antibody lowers BALF and plasma IL-33. Interleukin-33 levels in (**a**) BALF and (**b**) plasma in control, nano-TiO_2_ exposed groups and in rats pre-treated with an anti-IL-33 antibody. Rats were pre-treated with an anti-IL-33 antibody (1 mg/kg) 30 min prior to exposure. Plasma and BALF IL-33 levels were measured 4 h post-exposure. Statistics were analyzed with two-way ANOVA (*P* ≤ 0.05). * Sham control group vs. nano-TiO_2_ exposed groups, Ŧ *P* < 0.05 Exposed + Anti-IL-33 Antibody vs Exposed

IL-33 plays a significant role in initiating and modulating lung inflammatory and immunological responses [24], by inducing the secretion of Th2 pro-inflammatory cytokines. It is constitutively present in mucosal epithelial cells and acts as a “danger” signal after tissue injury by activating immune cells. To determine the effect of nano-TiO_2_ on pulmonary and plasma IL-33 levels, immunohistochemistry of tracheal sections was conducted 4 h post-exposure. In a separate cohort of control and exposed animals, multiplex analysis of plasma and BALF IL-33 was also performed 4 h post-exposure. Figure 4 shows representative images of lung sections from control and exposed rats stained for IL-33 4 h post-exposure and the quantitative measurements for both groups. Relative fluorescence intensity of IL-33 was significantly increased in exposed animals by 26.8 ± 4.3%, while plasma and BALF levels indicated a 37.87 ± 2.3% and 171.26 ± 13% increase in IL-33 respectively **(**Fig. 5**)**. Interestingly, treatment 1 h prior to exposure to nano-TiO_2_ with pharmacological grade polyclonal anti-IL-33 antibody (EMD Millipore, Temecula, CA: intraperitoneal 1 mg/kg) resulted in a decrease in plasma and BALF IL-33 levels 4 h post-exposure (61.1 ± 2.1% in plasma and 149.7 ± 7% in BALF) when compared to nano-TiO_2_ exposed and untreated animals. The results herein described provide evidence that reveals the critical role played by IL-33 in the initiation of the inflammatory response to ENM.

### Flow cytometric analysis of ILC1 and ILC2

**Fig. 6:**
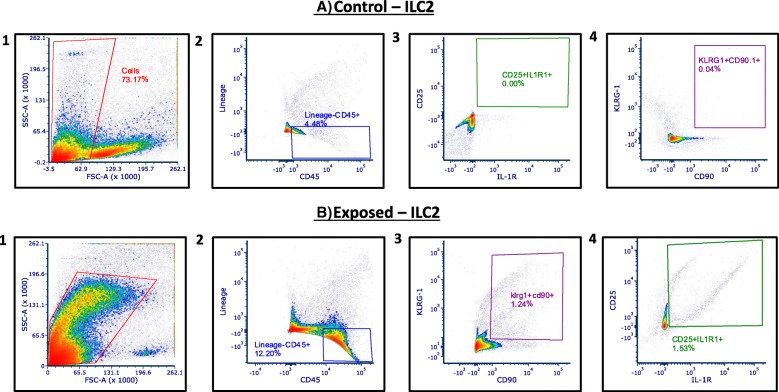
Flow cytometric analysis of lung-resident Group II innate lymphoid cells. Flow cytometry of lung tissue 4 h post-exposure for Group II Innate Lymphoid Cells in (**a**) control and (**b**) nano-TiO_2_ exposed animals (*N* = 6)

**Fig. 7:**
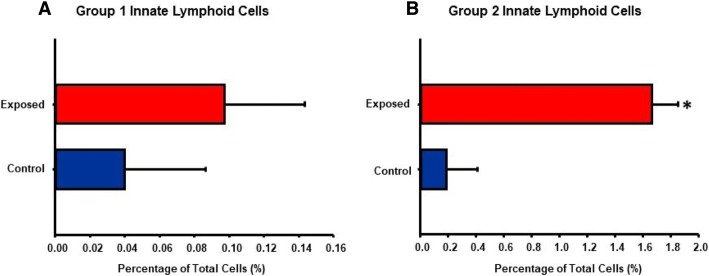
Nano-TiO_2_ exposure increases pulmonary ILC2 but not ILC1. Quantification of percentage of total pulmonary cells of (**a**) ILC1 and (**b**) ILC2 in control and nano-TiO_2_ exposed animals (*N* = 6). Statistics were analyzed with two-way ANOVA (*P* ≤ 0.05), * Sham control group vs. nano-TiO_2_ exposed groups

Polarization of T-cells requires several days [25], therefore, the early appearance of Th2 cytokines in the circulation after nano-TiO_2_ suggests that other, more rapid immune effector cells may be involved. Previous work has indicated that both ILC1 and ILC2 [26, 27] reside in the lungs and may be involved in lung inflammatory responses and pathology. Therefore, we next wanted to determine the effect of nano-TiO_2_ exposure on ILC1 and ILC2 levels in the lungs. For this reason, multi-parametric flow cytometric analysis was conducted on lung tissue from control and nano-TiO_2_ exposed Sprague-Dawley rats for ILC1 *(Data not shown)* and ILC2 **(**Fig. 6**)** 4 h post-exposure**.** No significant differences were noted in ILC1 levels pre- and post- exposure while ILC2 levels increased from 0.19 ± 0.22% to 1.66 ± 0.19% **(**Fig. 7a and b).

### NF-ĸB phosphorylation and Th2 cytokine secretion by isolated ILC2

**Fig. 8:**
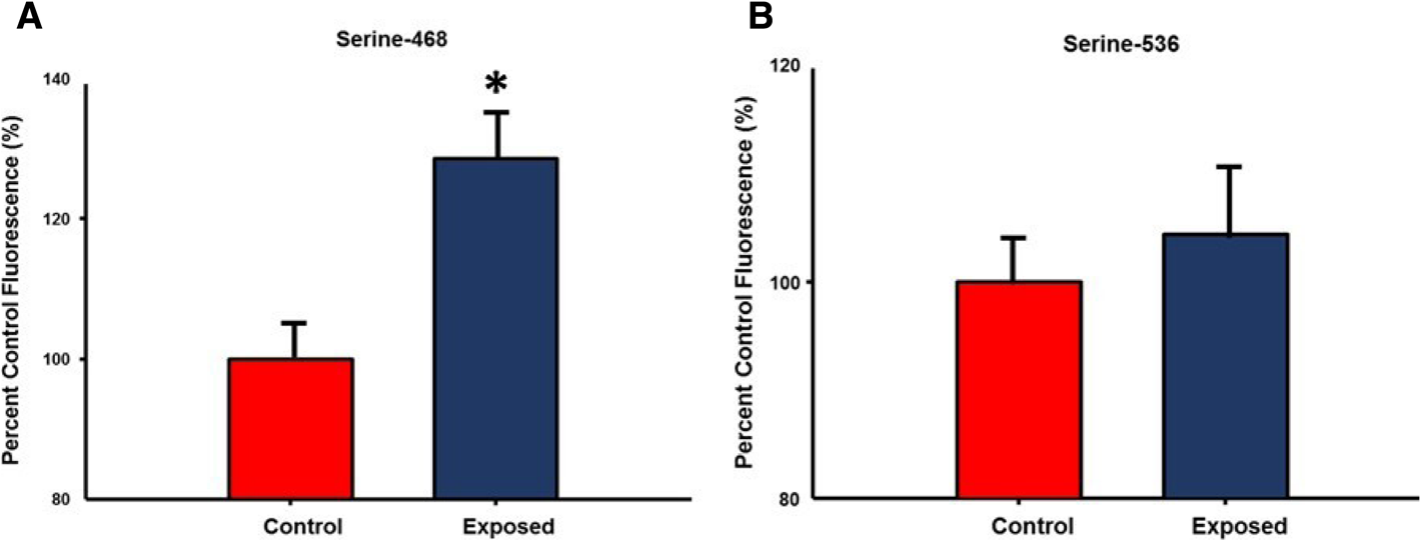
Nano-TiO_2_ exposure increases NF-ĸB phosphorylation at Serine-468 in isolated ILC2. Quantification of NF-ĸB phosphorylation at (**a**) serine-468 and (**b**) serine 536 in isolated ILC2 from isolated. ILC2 from control and nano-TiO_2_ exposed animals (N = 6). Statistics were analyzed with two-way ANOVA (P ≤ 0.05), * Sham control group vs. nano-TiO_2_ exposed groups

Activation of the NF-κB pathway has been shown to play a role in inflammation through its ability to induce the transcription of proinflammatory genes [28, 29]. Specifically, phosphorylation of NF—ĸB at both serine residues 468 and 536 are associated with inflammatory response [29, 30]. Therefore, levels of phospho- NF—ĸB were measured in isolated ILC2 from control and nano-TiO_2_ exposed animals **(**Fig. 8**)**. No significant differences were noted in phosphorylation of the Serine-536 residue between the 2 groups, while phosphorylation of Serine-468 was increased after acute pulmonary nano-TiO_2_ exposure (128.6 ± 7.24% of control levels).

**Fig. 9:**
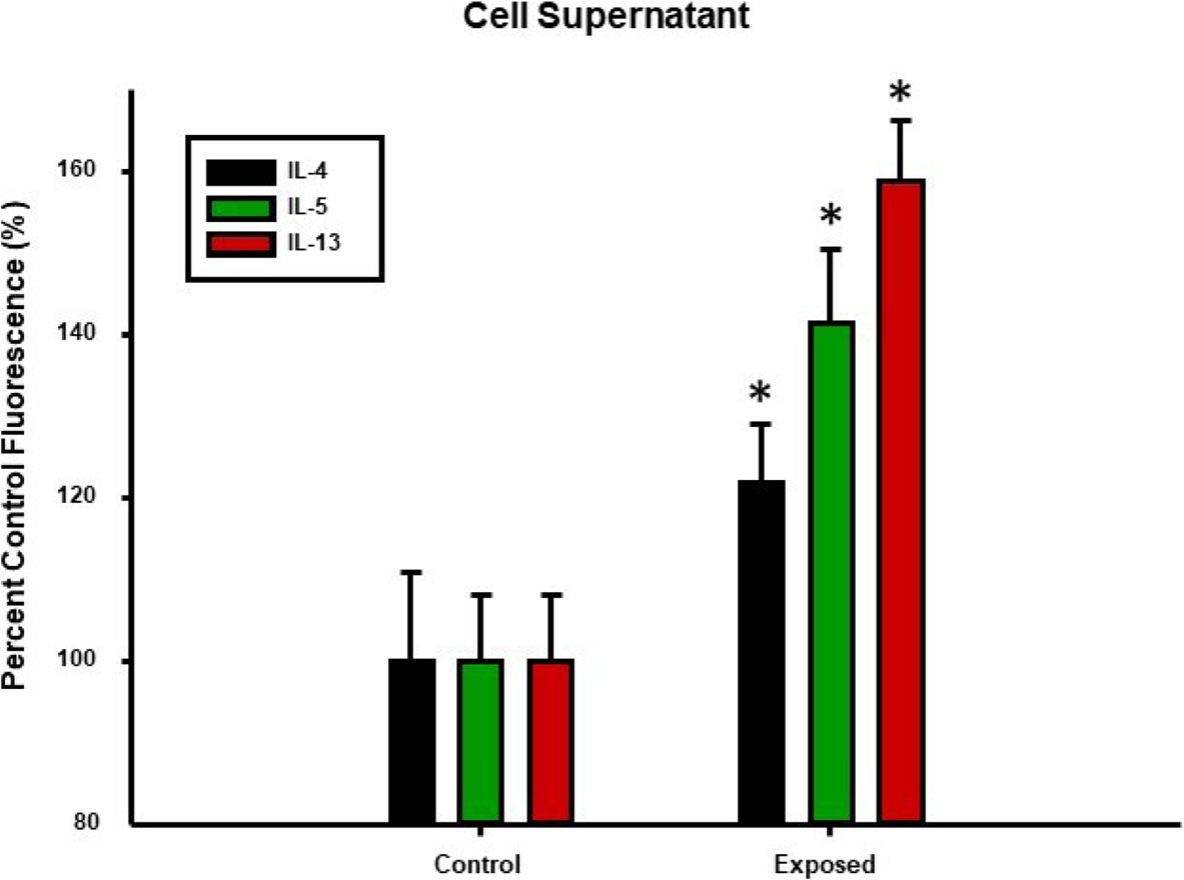
Th2 cytokine secretion in isolated ILC2 is increased by nano-TiO_2_ exposure. Multiplex cytokine analysis showing concentrations of IL-4, IL-5, IL-13 supernatant from isolated ILC2 cultured overnight from control and nano-TiO_2_ exposed animals (N = 6). Statistics were analyzed with two-way ANOVA (*P* ≤ 0.05), * Sham control group vs. nano-TiO_2_ exposed groups

Lastly, isolated ILC2 were cultured overnight and IL-4, IL-5 and IL-13 levels in the cell-culture media were measured. Nano-TiO_2_ resulted in a significant increase in IL-4, IL-5, and IL-13 (122 ± 7.2%, 141.4 ± 9.1%, 158.8 ± 7.6% of control levels respectively) **(**Fig. 9**)**.

### Effect of acute pulmonary Nano-TiO_2_ exposure and systemic immunotherapy on uterine radial arterioles

#### Endothelium-dependent dilation

In order to identify the acute reproductive effects associated with nano-TiO_2_ exposure, radial arterioles were isolated from the uterus of control and exposed animals 24 h post-exposure and endothelium-dependent and independent dilation along with adrenergic sensitivity were tested. Previous work by our group has shown that ENM exposure impacts vascular reactivity most severely within 24 h, a condition which has been shown to improve but not fully return to control levels after 168 h [30]. Based on these findings, all vascular assessments for this study were conducted 24 h post-exposure.

**Fig. 10:**
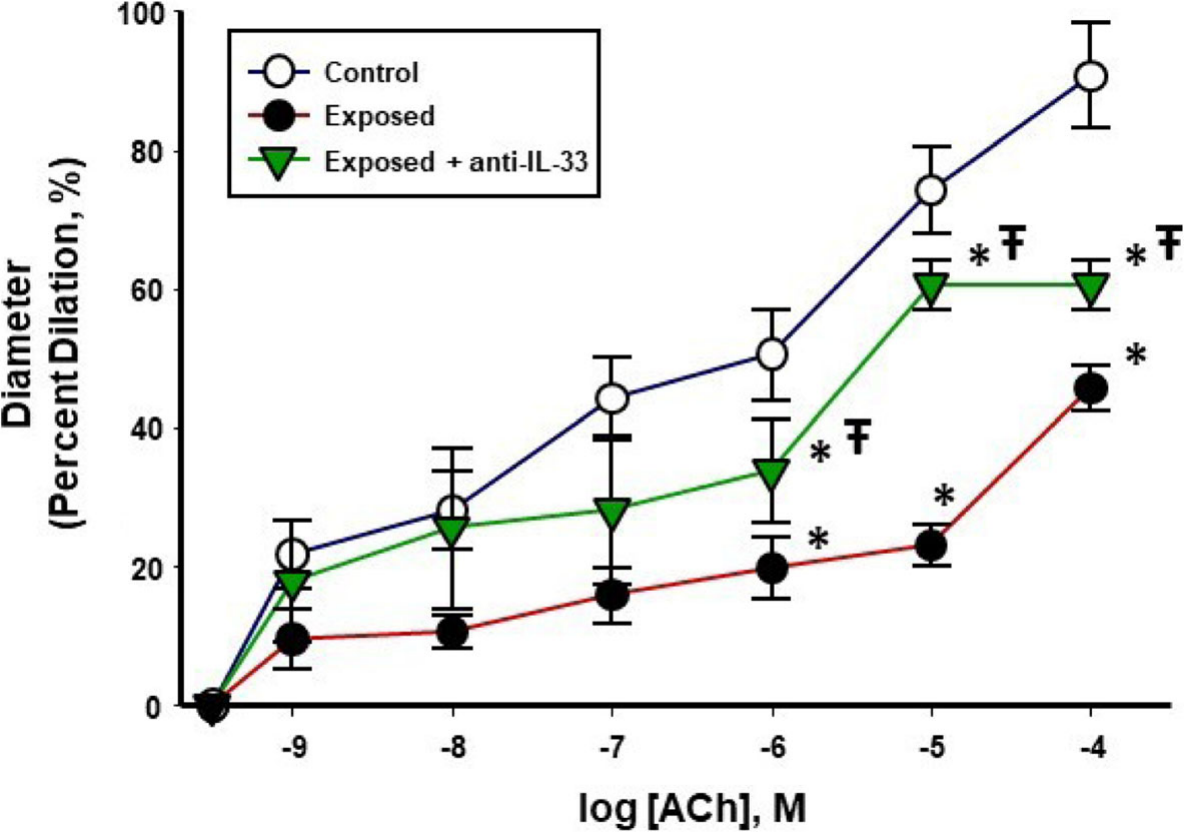
Endothelium-dependent dilation of radial arterioles is blunted by nano-TiO_2_ exposure and improved by pre-treatment with an anti-IL-33 antibody. Endothelium-dependent dilation of uterine radial arterioles from control, exposed and anti-IL-33 antibody treated animals was determined using pressure myography (*n* = 12–18). All vascular assessments were performed 24 h post-exposure. Statistics were analyzed with two-way ANOVA (*P* ≤ 0.05). * Sham control group vs. nano-TiO_2_ exposed groups, Ŧ *P* < 0.05 Exposed + Anti-IL-33 Antibody vs Exposed

Endothelium-dependent dilation of radial arterioles was significantly impaired following intratracheal instillation of 100 μg of nano-TiO_2_**(**Fig. 10**)**, with a mean decrease in dilation of 49.23 ± 6.5% compared to controls and 24.35 ± 10% compared to anti-IL-33 antibody-treated rats. These results suggest that pulmonary nano-TiO_2_ exposure disrupts normal physiological vascular endothelial function and that pre-treatment with an anti-IL-33 antibody partially attenuates the systemic acute inflammatory cascade triggered by lung-derived IL-33.

In contrast to endothelium-dependent relaxation, no point to point differences were seen in α-adrenergic and endothelium-independent dilation between control, nano-TiO_2_ exposed, and treated groups *(Data not shown)*.

## Discussion

The inflammatory paradigm tested herein has been suggested by previous studies [16, 31, 32]. While these studies explored pulmonary responses to exogenously administered IL-33, carbon nanotubes and viral infection, our findings provide additional evidence of a similar link between acute pulmonary ENM exposure and subsequent inflammatory mechanisms with systemic microvascular consequences. Perhaps the most important finding of this study is the identification of IL-33 as a potential contributor to the systemic inflammatory phenotype and microvascular dysfunction resulting from acute pulmonary ENM exposure.

We hypothesized that the innate response to pulmonary nano-TiO_2_ exposure is triggered by the secretion of IL-33 by lung epithelial cells. IL-33, a member of the IL-1 family, plays a significant role in initiating and modulating lung inflammatory and immunological responses [33], by inducing the secretion of Th2 pro-inflammatory cytokines through the Suppression of Tumorigenicity 2 (ST2) receptor in a variety of immune cells [32, 34]. Previous studies have shown that exposure to multi-walled carbon nanotubes triggers the IL-33/ST2 axis leading to the activation of mast-cells and macrophages [20]. Interestingly, IL-33 is also required for the maturation, activation and egress of ILC2 lineage cells from the bone marrow to specific target organs [35]. Several previous studies by our group and others have extensively investigated the cardiovascular effects of pulmonary ENM exposure [36, 37]. These studies consistently reveal that nano-TiO_2_ exposure increases leukocyte activation [38], proinflammatory mediators [39], and oxidative stress [40]. Collectively, this results in endothelium-dependent dysfunction. ILCs are a recently identified subpopulation of immune effector cells [33, 41]. They are critical, non-cytolytic tissue-resident cells that can be activated and respond acutely to danger signals or “alarmins” such as IL-33 from mucosal tissue by producing a distinctive array of cytokines to maintain mucosal integrity. Group II ILCs specifically, secrete the cytokines IL-5, IL-13, IL-9, amphiregulin, and low quantities of IL-4 [31] and have been linked to several lung-associated conditions including pathogen infections (viruses and helminths), asthma, and pulmonary fibrosis [42]. Lung ILC2 have an important role in regulating tissue remodeling and repair during acute epithelial injury and asthma [43]. Considered together, it is reasonable to speculate that the IL-33/ILC2 axis may impact systemic microvascular function after pulmonary ENM exposure.

Perhaps, the most significant finding of this study is the identification of a potential role for IL-33 in the initiation of an acute inflammatory response and the effects on systemic microvascular dysfunction **(**Fig. 10**)**. This was achieved by interrupting the IL-33/ST2 signaling axis with an anti-IL-33 antibody treatment prior to ENM exposure. In these experiments, the control group (IT saline) did not receive the anti-IL-33 antibody. The scientific rationale for this decision was that the technique (isolated, perfused and pressurized arterioles) used to assess microvascular dysfunction is a plasma free system. As such, it was impossible to assess the role of the complete IL-33/ST2 axis on arteriolar tone and/or reactivity. Whereas, this approach directly measured the impact that IL-33 inflammatory signaling has on microvascular function. It remains a distinct possibility that the IL-33/ST2 axis activated by pulmonary ENM exposure directly influences microvascular tone and/or reactivity. IL-33 expression has been identified in human vascular endothelial cells; whereas, is absent in murine vascular tissue [44]. This supports our postulate that IL-33 is impacting microvascular function via inflammation, rather than a direct activation of the ST2 receptor. Future in vivo studies must directly assess the impact of circulating IL-33 on microvascular tone, reactivity and blood flow.

While our study provides plausible mechanistic insights into both the inflammatory and microvascular effects associated with ENM exposure, additional considerations as well as the limitations must be kept in mind. ENM exposure has been shown to affect other innate immune effector cells such as neutrophils [45, 46], macrophages [47, 48], and dendritic cells [49]. Therefore, other mechanisms including local generation of reactive of oxygen species, secretion of chemokines and prostaglandins by macrophages and stimulation of the adaptive immune system by antigen-presenting cells may play a role in the systemic inflammatory response and vascular effects seen in this study. It is also worth noting that in this study an immune response associated with pulmonary ENM exposure was observed as early as 4 h post-exposure, as evidenced not only by an increase in the proportion of lung-resident ILC2s **(**Fig. 7**)**, but also by augmented levels of the Th2 cytokines IL-4, IL-5, and IL-13 **(**Figs. 2 and 3**)**. This response timeframe is in agreement with previous studies, that have reported IL33, ILC2 numbers as well as lung and serum Th2 cytokines increase within 6–12 h of lung injury [50, 51]. Furthermore, isolated ILC2s have been shown to respond dose-dependently to transient or continuous stimulation with IL-2 and/or IL-33 within 3 h [52]. Similarly, nano-TiO_2_ instillation at lung burdens of 40–160 μg/rat has also been shown to increase BALF inflammatory mediators within 4 h of exposure [53]. We report herein a similar phenomenon as the lung burden used in this study was comparable at 100 μg/rat.

Our findings contribute consistent evidence for a role of ILC2s in the acute pulmonary response to ENM exposure. Due to the inherent limitations associated with our experimental in vivo model, we were unable to directly establish causality. In order to directly confirm such an observation, ILC2 deletion would be necessary. Our research program has a long-standing interest in the systemic microvascular consequences of pulmonary particle exposure. Rat models are primarily used in our in vivo studies as they are the most appropriate and widely used animals for microvascular research. However, they are not commonly or easily adapted for transgenic studies. As such, an ILC2 rat deletion model does not exist. If such a model were created in the future, it would then be possible to directly confirm a causative relationship between ENM exposure and ILC2 activation, proliferation and recruitment.

Lastly, to allow periodic blood sampling for cytokine analysis, intratracheal instillation was chosen as the preferred exposure method. The above-mentioned dose of nano-TiO_2_ was selected based on previous studies conducted by our group which have shown that it is associated with significant microvascular impairment, oxidative and nitrosative stress and alveolar macrophage recruitment [36, 54]. Additionally, to better understand the relevance of the exposure paradigm used in this study to humans, alveolar surface areas must be known [55]. The rat alveolar surface area is 0.4 m^2^/lung. Therefore, the rat burden of 50 μg/lung would result in 125 μg/m^2^. Given that the human alveolar surface area is 102 m^2^, the equivalent human burden of this exposure paradigm would be 12.75 mg. The next logical question is: How long would it take to achieve this burden in humans? In this regard, lung burden may be calculated as:

$$ nano\hbox{-} {TiO}_2 aerosol\ concentration\cdot \kern0.5em minute\ ventilation\cdot exposure\ duration\ deposition\ fraction, $$

with the following values:

$$ 25.5\  mg= nano\hbox{-} {TiO}_2 aerosol\ concentration\cdot 7600\  ml/\mathit{\min}\ \left( 8\  hrh/ day\cdot 60\ \mathit{\min}/ hr\right)\cdot 14\%, $$

and therefore:

$$ 25.5\  mg= nano\hbox{-} {TiO}_2 aerosol\ concentration\cdotp 0.51\ {m}^3/ day. $$

Considering both the National Institute for Occupational Safety and Health (NIOSH) and the Occupational Safety and Health Administration (OSHA) Permissible Exposure Limit (0.3 mg/m^3^ and 5 mg/m^3^ respectively) [4], it would require 0.34 working years or 122 working days for a human to achieve similar exposure levels to those used in this study.

## Conclusion

In conclusion, the current study provides novel evidence that links acute pulmonary ENM exposure and systemic microvascular effects. Because this inflammatory link exists between the lung and the uterine microcirculation, it remains to be determined if such an axis extends to the placenta and or fetus.

## Materials and methods

### Nanomaterial characterization

Nano-TiO_2_ P25 powder, obtained from Evonik (Aeroxide TiO_2_, Parsippany, NJ), has previously been shown to be a mixture composed primarily of anatase (80%) and rutile (20%) TiO_2_, with a primary particle size of 21 nm and a surface area of 48.08 m^2^/g [54], and a Zeta-potential of − 56.6 mV [56]. Elemental composition analysis of the nano-TiO_2_ suspension was conducted via energy dispersive spectroscopy.

Scanning electron microscopy was performed by diluting the nano-TiO_2_ 1:100 with filtered distilled water. 0.5 ml of the diluted particle solution was filtered onto a 0.2 μm polycarbonate filter. A wedge-shaped portion of the polycarbonate filter was mounted onto carbon double stick tape which was affixed to a 13 mm aluminum stub. The sample was sputter coated with gold-palladium for two minutes. The sample was imaged using a Hitachi S4800 field-emission scanning electron microscope (Tokyo, Japan).

### Experimental animals and exposure

Female (8–10 weeks) Sprague – Dawley rats were purchased from Hilltop Laboratories (Scottdale, PA) and housed in an AAALAC approved facility at West Virginia University (WVU) with 12:12 h light – dark cycle and regulated temperature. Rats were allowed ad libitum access to food and water. All procedures were approved by the Institutional Animal Care and Use Committee of WVU.

100 μg of nano-TiO_2_ were suspended in 250 μL of vehicle (Normosol and 5% fetal bovine serum) for intratracheal instillation (IT) 24 h prior to experimentation. Nano-TiO_2_ suspensions were sonicated for 1 min to ensure homogenous distribution of nanoparticles. Rats were anesthetized using 5% isoflurane and placed on a mounting stand. 250 μL of the nano-TiO_2_ suspension was then instilled intratracheally. Sprague - Dawley rats instilled with 250 μL of vehicle were used as controls.

### Mean arterial pressure (MAP) acquisition

Rats were anesthetized with isoflurane gas (5% induction, 2–3.5% maintenance). The animals were placed on a heating pad to maintain a 37 °C rectal temperature. The trachea was intubated to ensure an open airway and the right carotid artery was cannulated to acquire mean arterial pressure (MAP). The MAP was measured via a pressure transducer and recorded by PowerLab830 (AD Instruments, Colorado Springs, CO).

### Multiplex cytokine panels of serum and Bronchoalveolar lavage fluid

Whole blood (1–2 ml) was collected via tail vein puncture from exposed and control animals at time 0, 1 h, 2 h, and 4 h after exposure into EDTA vacutainers and centrifuged (1000 x g) to collect plasma which was flash-frozen in liquid nitrogen and stored at − 80°C until analysis. Rats were euthanized and tissues harvested for further analysis. Multi-spot inflammatory assays were completed per manufacturer’s directions (Meso Scale Diagnostics, Rockville, MD) for: lipocalin-2, TSP-1, TIMP-1, MCP-1, interferon (IFN-γ), interleukin (IL)-1β, IL-4, IL-5, IL-6, KC/GRO, IL-10, IL-13, and TNF-α.

### Immunohistochemistry

Gelatin-coated cover slips of serial tissue sections were incubated with anti-rabbit (1:100) IL-33 (Cloud-Clone Corp., Katy, TX) overnight at 4°C, followed by 3 five-minute washes with cold PBS with 0.1% Triton-X. The sections were then incubated with a FITC conjugated goat anti-rabbit (1:100, Invitrogen, Carlsbad, CA) at 37°C for one hour, followed by 3 five-minute washes with cold PBS with 0.1% Triton-X. Cover slips were then mounted on slides for visualization on a Zeiss fluorescent microscope (Zeiss, Thornwood, NY).

### Preparation of cell suspensions

Lungs were perfused with sterile PBS by injection into the right ventricle to remove remaining blood and then placed in PBS containing 20 mg/ml Collagenase A, 2.4 U/ml Dispase II solution, and 50 μl/ml DNAse (Roche, Indianapolis, IN) for 30 min.

The lung tissue was dissociated using the gentleMACS Octo Dissociator (Miltenyi Biotec, Auburn, CA) and then centrifuged (1100 x g for 10 min) to collect the sample material. The sample was resuspended with PBS and used for magnetic cell separation and flow cytometry.

### Flow cytometry

#### Antibodies and reagents

ILC1 and ILC2 numbers 4 h after exposure to nano-TiO_2_ from lung tissue were determined by flow cytometry as previously described [57–59]. Briefly, ILC1 were defined as Lin-CD45 + CD161 + CD335+, while ILC2 were defined as Lin-CD45 + CD90.1 + KLRG-1 + CD25 + IL1R1 + .

Monoclonal antibodies specific for CD90.1 (FITC), KLRG1 (APC EFLUOR 780), CD25 (APC), CD161 (PERCP EFLUOR 710), and CD335 (EFLUOR 450) were purchased from eBioscience (San Diego, CA). Lineage cocktail (Lin; ALEXA FLUOR 700) was used to gate out (CD3, CD14, CD16, CD19, CD20, and CD56: Biorad, Hercules, CA) CD3 T lymphocytes, CD14 Monocytes, CD16 NK cells, granulocytes, CD19 B lymphocytes, CD20 B lymphocytes, and CD56 NK cells. Antibodies for CD45 (Cyanine 5) were from Invitrogen (Carlsbad, CA), while those for IL1R1 (PE) where from Sino Biologicals (North Wales, PA). Flow cytometry was performed on a FACSAria (BD Bioscience, Franklin Lakes, NJ). Data were analyzed with FCS Express 6 Software (De Novo Software, Glendale, CA).

#### Isolation of group II innate lymphoid cells

ILC2 were isolated as per manufacturer’s instructions (Miltenyi Biotec, Auburn, CA). Briefly, lineage-positive cells were indirectly labeled with a cocktail of ALEXA FLUOR 700-conjugated antibodies, as primary labeling reagent, and antibodies conjugated to MicroBeads were used as secondary labeling reagents. In the second step, lineage-negative cells were labeled with CD45, CD90.1, KLRG-1, CD25, and IL1R1, labeled with MicroBeads and isolated by positive selection from the pre-enriched lineage negative cell-fraction by separation over a MACS Column (Miltenyi Biotec, Auburn, CA), which was placed in the magnetic field of a MACS Separator. After negative selection, the cells were subsequently eluted as the positively selected cell fraction containing ILC2.

#### Cytokine analysis of cell culture media and measurement of Phospho-NF-ĸB

Isolated ILC2s were incubated (37 °C, 90% humidity, 5% CO_2_) overnight in supplemented (10% fetal bovine serum, 1% penicillin/streptomycin, 1% sodium pyruvate, 1% L-glutamine) Dulbecco’s Modified Eagle Medium (Corning, Manassas, VA). The following day, the cell culture medium and cellular portion were separated via centrifugation (1100 x g for 10 min). IL-4, IL-5, and IL-13 levels were measured in the cell culture medium via multi-spot inflammatory assays per manufacturer’s directions (Meso Scale Diagnostics, Rockville, MD). Lastly, isolated cells were lysed and phospho-NF-κB (Ser468 and Ser536) was measured (Meso Scale Diagnostics, Rockville, MD).

#### Systemic treatment with anti-Interleukin-33 antibody

Rats were treated 1 h prior to pulmonary nano-TiO_2_ exposure with pharmacological grade polyclonal anti-IL-33 antibody (CAT#ABF108, EMD Millipore, Temecula, CA: intraperitoneal 1 mg/kg). The antibody and dosage were selected based on previous murine studies [60]. The antibody used was a polyclonal anti-IL-33 antibody developed in the rabbit with specific reactivity toward mouse IL-33 and expected cross reactivity due to the close homology to rat IL-33. We determined the interspecies homology of the anti-IL-33 target between rats and mice to be 87% (Additional file 1: Figure S1). Plasma, bronchoalveolar lavage fluid, and lung tissue were obtained 4 h post-exposure from treated animals for measurement of IL-33. Uterine microvascular assessments were conducted 24 h after treatment in a separate cohort of animals.

#### Pressure Myography microvessel preparation

After measuring MAP, the uterus was removed and placed in a dissecting dish with physiological salt solution (PSS) maintained at 4 °C. Radial arterioles were isolated, transferred to a vessel chamber, cannulated between two glass pipettes, and tied with silk sutures in the chamber (Living Systems Instrumentation, Burlington, VT). The chamber was superfused with fresh oxygenated (5% CO_2_/21% O_2_) PSS and warmed to 37 °C. Arterioles were pressurized to 60 mmHg using a servo control system and extended to their in situ length. Internal and external arteriolar diameters were measured using video calipers (Colorado Video, Boulder, CO).

#### Arteriolar reactivity

Previous work by our group has shown that ENM exposure impacts endothelium-dependent dilation most severely within 24 h, a condition which has been shown to improve but did not fully return to control levels after 168 h. Based on these findings, all vascular assessments for this study were conducted 24 h post-exposure [30].

Arterioles were allowed to develop spontaneous tone, defined as the degree of constriction experienced by a blood vessel relative to its maximally dilated state. Vascular tone ranges from 0% (maximally dilated) to 100% (maximal constriction). Vessels with a spontaneous tone ≥20% less than initial tone were included in this study. After equilibration, various parameters of arteriolar function were analyzed. Vessels that did not develop sufficient spontaneous tone were not included in the data analysis.

- **Endothelium-Dependent Dilation**— arterioles were exposed to increasing concentrations of acetyl choline (ACh: 10^− 9^ - 10^− 4^ M) added to the vessel chamber.
- **Endothelium-Independent Dilation**—increasing concentrations of sodium nitroprusside (SNP: 10^− 9^ - 10^− 4^ M) were used to assess arteriolar smooth muscle responsiveness.
- **Arteriolar Vasoconstriction**—arterioles were exposed to increasing concentrations of phenylephrine (PE: 10^− 9^ - 10^− 4^ M). The steady state diameter of the vessel was recorded for at least 2 min after each dose. After each dose curve was completed, the vessel chamber was washed to remove excess chemicals by carefully removing the superfusate and replacing it with fresh warmed oxygenated PSS. After all experimental treatments were complete, the PSS was replaced with Ca^2+^-free PSS until maximum passive diameter was established.

### Pressure Myography calculations

Data are expressed as means ± standard error. Spontaneous tone was calculated by the following equation:

$$ Spontaneous\ tone\ \left(\%\right)=\left\{\frac{\left( Dm- Di\right)}{Di}\right\}x\ 100 $$

, where Dm is the maximal diameter and Di is the initial steady state diameter recorded prior to the experiment. Active responses to pressure were normalized to the maximal diameter using the following formula:

$$ Normalized\ diameter= Dss/ Dm $$

, where Dss is the steady state diameter recorded during each pressure change. The experimental responses to ACh, PE, and SNP are expressed using the following equation:

$$ Diameter\ \left( percent\ maximal\ diameter\right)=\left\{\frac{\left( Dss- Dcon\right)}{\left( Dm- Dcon\right)}\right\}x\ 100 $$

, where DCon is the control diameter recorded prior to the dose curve, DSS is the steady state diameter at each dose of the curve. The experimental response to PE is expressed using the following equation:

$$ Diameter\ \left( percent\ maximal\ diameter\right)=\left\{\frac{\left( Dcon- Dss\right)}{(Dcon)}\right\}x\ 100 $$

Wall thickness (WT) was calculated from the measurement of both inner (ID) and outer (OD) steady state arteriolar diameters at the end of the Ca^2+^ free wash using the following equation:

$$ WT=\left( OD- ID\right)/2 $$

Wall-to-lumen ratio (WLR) was calculated using the following equation:

$$ WLR= WT/ ID $$

### Statistics

Point-to-point differences in the dose response curves were evaluated using two-way repeated measures analysis of variance (ANOVA) with a Tukey’s *post-hoc* analysis when significance was found. The slopes of the dose response curves were determined through a nonlinear regression. The animal characteristics, vessel characteristics and dose response curve slopes were analyzed using a one-way ANOVA with a Tukey *post-hoc* analysis when significance was found. All statistical analysis was completed with GraphPad Prism 5 (San Diego, CA) and SigmaPlot 11.0 (San Jose, CA). Significance was set at *p* < 0.05, n is the number of arterioles, and N is the number of animals.

## Additional file

Additional file 1: **Figure S1.** IL-33 sequence homology. Multiple sequence alignment of the protein sequence of IL-33 in mice (query) and rats (subject) showing the significant interspecies homology of murine IL-33. (ZIP 259 kb)

## Abbreviations

ACh: Acetylcholine
ANOVA: Analysis of variance
APC: Allophycocyanin
BALF: Broncho alveolar lavage fluid
CD: Cluster of differentiation
Dcon: Control diameter
Di: Initial diameter
Dm: Maximal diameter
Dss: Steady state diameter
EDTA: Ethylenediaminetetraacetic acid
ENM: Engineered nanomaterials
FITC: Fluorescein isothiocyanate
ID: Inner diameter
IFN-γ: Interferon-gamma
IL: Interleukin
ILC: Innate lymphoid cells
KC/GRO: Keratinocyte chemoattractant/human growth-regulated oncogene
KLRG-1: Killer cell lectin-like receptor subfamily G member 1
Lin: Lineage
MCP-1: Monocyte chemoattractant protein 1
Nano-TiO_2_: Titanium dioxide nanoparticles
NF-ĸB: Nuclear factor kappa-light-chain-enhancer of activated B cells
NIOSH: National Institute for Occupational Safety and Health
NK: Natural Killer
NO: Nitric oxide
OD: Outer diameter
OSHA: Occupational Safety and Health Administration
PBS: Phosphate buffered saline
PE: Phenylephrine
PE: Phycoerythrin
SEM: Scanning electron microscope
SEM: Standard error of the mean
Ser-468: Serine-468
SNP: Sodium nitroprusside
ST2: Suppressor of tumor tumorigenicity
Th2: T-helper type 2
TIMP-1: Tissue inhibitor of metalloproteinases 1
TNF- α: Tumor necrosis factor alpha
TSP-1: Thrombospondin 1
WLR: Wall-to-lumen ratio
WT: Wall thickness
