# Supplementary material for: Group II innate lymphoid cells and microvascular dysfunction from pulmonary titanium dioxide nanoparticle exposure
Source: Part Fibre Toxicol. 2018 Nov 9;15:43. doi: 10.1186/s12989-018-0280-2 (PMC6230229; doi:10.1186/s12989-018-0280-2)
Supplement: Supplementary file 1 — Figure S1. IL-33 sequence homology. Multiple sequence alignment of the protein sequence of IL-33 in mice (query) and rats (subject) showing the significant interspecies homology of murine IL-33. (ZIP 259 kb) [file 12989_2018_280_MOESM1_ESM.zip › Supplementary Figure 1.pdf]

Supplementary Figure 1

Score	Expect	Method	Identities	Positives	Gaps
468 bits(1203)	7e-174	Compositional matrix adjust.	232/266(87%)	244/266(91%)	2/266(0%)

```
Query  1    MRPRMKYSNSKISPAKFSSTAGEALVPPCKIRRSQQKTKEFCHVYCMRLRSGLTIRKETS  60
            MRPRMKYSNSKISPAK  ST+G ALVPPCKI RSQQKTK+ CHVYCMRLRSGLTIRKET
Sbjct  1    MRPRMKYSNSKISPAKCDSTSGRALVPPCKITRSQQKTKDICHVYCMRLRSGLTIRKETC  60

Query  61   YFRKEPTKRYSLKSGTKHEENFSAYPRDSRKRSLLGSIQAFAASVDTLSIQGTSLLTQSP  120
            YF KEP KRYSLKSG+KHE   S    DSRKRSLLGSIQAFAASVDTLSIQGTSLLT+S
Sbjct  61   YFGKEPAKRYSLKSGSKHEGRLSTCLPDSRKRSLLGSIQAFAASVDTLSIQGTSLLTESC  120

Query  121  ASLSTYNDQSVSFVLENGCYVINVDDSGKDQEQDQVLLRYYESPCPASQSGDGVDGKKLM  180
            A LSTYNDQSVSFVLENGCYVINV+D GK+QE+D+VLLRYYES  PA QSGDGVDGKKLM
Sbjct  121  A-LSTYNDQSVSFVLENGCYVINVEDCGKNQEKDKVLLRYYESSFPA-QSGDGVDGKKLM  178

Query  181  VNMSPIKDTDIWLHANDKDYSVELQRGDVSPPEQAFFVLHKKSSDFVSFECKNLPGTYIG  240
            VNMSPIKDTDIWL+ANDKDYSVELQ+GDVSPP+QAFFVLHKKSSDFVSFECKNLPGTYIG
Sbjct  179  VNMSPIKDTDIWLNANDKDYSVELQKGDVSPPDQAFFVLHKKSSDFVSFECKNLPGTYIG  238

Query  241  VKDNQLALVEEKDESCNNIMFKLSKI  266
            VKDNQLALVEE DESCNNIMFKLSK+
Sbjct  239  VKDNQLALVEENDESCNNIMFKLSKM  264
```

	Max Query	Total Query	Query Cover	E value	Identity
Interleukin-33	466	466	100%	7e-174	87%
