# Supplementary material for: Group II innate lymphoid cells and microvascular dysfunction from pulmonary titanium dioxide nanoparticle exposure
Source: Part Fibre Toxicol. 2018 Nov 9;15:43. doi: 10.1186/s12989-018-0280-2 (PMC6230229; doi:10.1186/s12989-018-0280-2)
Supplement: Supplementary file 1 — Figure S1. IL-33 sequence homology. Multiple sequence alignment of the protein sequence of IL-33 in mice (query) and rats (subject) showing the significant interspecies homology of murine IL-33. (ZIP 259 kb) [file 12989_2018_280_MOESM1_ESM.zip › Supplementary Figure ILC2 Manuscript.docx]

**Supplementary Figure Legend**

**Supplementary Figure 1. IL-33 sequence homology.** Multiple sequence alignment of the protein sequence of IL-33 in mice (query) and rats (subject) showing the significant interspecies homology of murine IL-33.
